# NEIGHBORHOOD AFFLUENCE AND RISK OF INCIDENT DEMENTIA: ROLE OF PHYSICAL ACTIVITY DESTINATIONS

**DOI:** 10.1093/geroni/igad104.1186

**Published:** 2023-12-21

**Authors:** Kyle Moored, Michael Desjardins, Breanna Crane, Patrick Donahue, Parveen Garg, Andrea Rosso, Gina Lovasi, Michelle Carlson

**Affiliations:** Johns Hopkins Bloomberg School of Public Health, Baltimore, Maryland, United States; Johns Hopkins Bloomberg School of Public Health, Baltimore, Maryland, United States; Johns Hopkins Bloomberg School of Public Health, Baltimore, Maryland, United States; Johns Hopkins University, Baltimore, Maryland, United States; University of Southern California Keck School of Medicine, Los Angeles, California, United States; University of Pittsburgh, Pittsburgh, Pennsylvania, United States; Dornsife School of Public Health, Philadelphia, Pennsylvania, United States; Johns Hopkins Bloomberg School of Public Health, Baltimore, Maryland, United States

## Abstract

Neighborhood affluence encompasses the degree of high educational, occupational, and wealth concentration within residential communities. Highly affluent neighborhoods may reduce dementia risk in part by providing amenities that promote physical activity. We examined associations between neighborhood affluence, and specifically the quantity of nearby physical activity (PA) destinations (e.g., gyms), with incident dementia. Participants were 2,942 adults ≥65 years old from the Cardiovascular Health Cognition Study. Baseline neighborhood measures included 1) a 1990 Census-derived affluence index (percent with college education, percent with professional/managerial occupation, median home value) and 2) the count of PA destinations from the 1990 National Establishment Time Series (NETS) database that fell within a 1-km Euclidean buffer around the home. A total of 469 dementia cases (240 Alzheimer’s, 208 mixed/vascular, 21 other) were clinically adjudicated from neurological/neuropsychological examinations (Mean=5.3±1.8 follow-up years). In Cox proportional hazards models adjusted for clinic site and individual demographics (age, gender, race, marital status), each 1-SD higher neighborhood affluence was associated with 15% reduced hazard of all-cause dementia (HR=0.85, 95%CI: 0.77-0.94), largely driven by mixed/vascular subtype (HR=0.73, 95%CI: 0.64-0.86), but not Alzheimer’s subtype (HR=0.93, 95%CI: 0.81-1.07). Further, participants with ≥2 (versus no) nearby PA destinations had 37% reduced hazard of specifically mixed/vascular dementia (HR=0.63, 95%CI: 0.41-0.99). After also adjusting for individual health, income, and education, neighborhood affluence remained protective against mixed/vascular dementia (HR=0.77, 95%CI: 0.65-0.90), but number of PA destinations was no longer associated. Neighborhood affluence may reduce dementia risk via improved vascular health and operate through additional mechanisms beyond nearby PA amenities.

